# Effectiveness of combined regenerative medicine and exercise therapy for patients with knee osteoarthritis: a scoping review

**DOI:** 10.3389/fresc.2025.1612615

**Published:** 2025-07-22

**Authors:** Tomohiro Oka, Kosuke Suzuki, Katsuyoshi Tanaka, Kouhei Okuyama, Takashi Kitagawa

**Affiliations:** ^1^ Department of Physical Therapy, Osaka Health Science University, Osaka, Japan; ^2^ Graduate School of Health Sciences, Kobe University, Kobe, Hyōgo, Japan; ^3^ Department of Rehabilitation, Yamagata Saisei Hospital, Yamagata, Japan; ^4^ Department of Physical Therapy, Bukkyo University, Kyoto, Japan; ^5^ Department of Physical Therapy, School of Health Sciences, Shinshu University, Matsumoto, Japan

**Keywords:** scoping review, regenerative medicine, exercise therapy, knee osteoarthritis, pain relief, functional improvement

## Abstract

**Background:**

Regenerative therapies such as platelet-rich plasma (PRP) and stem cell treatments show promise for symptom relief in knee osteoarthritis (OA), but individual responses vary. Exercise therapy is a well-established intervention that enhances muscle strength and joint stability. Although both approaches are effective, their combined use remains underexplored. Notably, no systematic or scoping review has yet examined the specific types of regenerative medicine and exercise therapy used in combination, the outcome domains assessed (pain, function, structure, and quality of life), or the key evidence gaps. This scoping review aimed to examine these combinations, outcome domains, and gaps in the current evidence base.

**Methods:**

A literature search was conducted in MEDLINE, CENTRAL, and CINAHL using keywords including “knee osteoarthritis,” “regenerative medicine,” and “exercise therapy.” Studies were included if they compared regenerative medicine alone to regenerative medicine combined with exercise therapy. Two reviewers independently extracted data on pain, physical function, and patient-reported outcomes across short-term (6–24 weeks) and long-term (up to 96 weeks) follow-up.

**Results:**

Three studies [two randomized controlled trials (RCTs), one non-RCT] were included. Sample sizes ranged from 17 to 32. Despite variations in PRP type (pure vs. leukocyte rich), and exercise regimen (home-based vs. supervised), all studies showed significant advantages in pain and function for the combined intervention group. Benefits emerged as early as 6 weeks and persisted up to 96 weeks. One study also noted structural changes via ultrasound.

**Conclusions:**

Combining regenerative medicine with exercise therapy significantly enhances pain relief and functional outcomes in knee OA patients.

## Introduction

In knee osteoarthritis (OA), cartilage gradually deteriorates, resulting in pain, stiffness, and reduced functional ability, significantly impacting older adults' quality of life and contributing to disability (1–4). Globally, approximately 365 million people worldwide are affected by knee OA, highlighting its widespread impact (5). The increasing prevalence of knee OA owing to the aging population highlights the need for effective treatment strategies to manage symptoms and improve patient outcomes.

Traditional treatments for knee OA include conservative approaches such as pharmacotherapy, physical therapy, orthotic therapy, and surgical interventions such as total knee arthroplasty (TKA) (6). While TKA can alleviate symptoms and restore function, approximately 30% of the patients are reluctant to undergo surgery because of its invasive nature and potential complications (7). Recently, regenerative medicine has emerged as a promising alternative treatment for knee OA. Regenerative medicine involves the use of biological therapies, including platelet-rich plasma (PRP) and cell-based therapies, such as adipose-derived mesenchymal stem cells, to repair and regenerate damaged tissues (8, 9). Regenerative treatments aim to reduce pain and improve joint function by harnessing the natural healing processes. Previous studies have reported that regenerative medicine can achieve a 60% success rate in pain relief and knee function improvement (10). However, approximately 30%–40% of patients do not experience significant benefits, suggesting the need for adjunctive therapies to optimize treatment outcomes. Therefore, some studies have indicated that regenerative medicine alone is insufficient for reducing pain and improving physical function, highlighting the importance of combined treatment modalities (11, 12).

Exercise therapy is a well-documented intervention for knee OA, with systematic reviews recommending it for pain management and functional improvement (13, 14). Exercise therapy includes a wide variety of activities, such as aerobics, strength training, and flexibility exercises, to improve muscle strength, joint stability, and overall physical function. Combining exercise therapy with regenerative medicine may offer synergistic benefits, potentially improving the clinical outcomes in patients with knee OA undergoing regenerative medicine.

Exercise stimulates chondrocyte proliferation through mechanical loading and upregulates the expression of growth factors such as transforming growth factor-β and insulin-like growth factor-1, while regenerative medicine provides bioactive substances such as platelet-derived growth factor and vascular endothelial growth factor. These synergistic effects may accelerate cartilage repair and enhance anti-inflammatory processes (15, 16). Despite the potential advantages of this combined approach, comprehensive evidence summarizing its effectiveness remains limited. Preliminary searches in MEDLINE, the Cochrane Database of Systematic Reviews, and JBI Evidence Synthesis, yielded no systematic or scoping reviews on this topic.

This scoping review was conducted to systematically map the current evidence on the combination of regenerative medicine and exercise therapy in patients with knee OA. The review aims to clarify what types of regenerative therapies (e.g., platelet-rich plasma, stem cell therapy) and exercise modalities (e.g., strengthening, flexibility, aerobic) have been studied, what outcome domains have been assessed (e.g., pain, physical function, structural changes, and quality of life), and what gaps remain in the current research landscape.

## Materials and methods

A scoping review was conducted following the Preferred Reporting Items for Systematic Review and Meta-Analysis (PRISMA-ScR) (17) and JBI methodology for scoping reviews (18). An Open Science Framework protocol (https://osf.io/8uw6h/) was registered for review on July 11, 2024. Inclusion criteria were defined using participants, concepts, and a contextual framework.

### Eligibility criteria

This scoping review included patients with knee osteoarthritis who underwent treatment with regenerative medicine, without restrictions on age, sex, comorbidities, or Kellgren-Lawrence (KL) grade. Regenerative medicine therapies considered were pure, leukocyte-poor, and leukocyte-rich PRP; Autologous Protein Solution; stem cell therapy (Bone Marrow, Adipose, and Umbilical Cord Blood-derived mesenchymal stem cells); growth factor injections; gene therapy; tissue engineering; exosome therapy; and prolotherapy. Observational and interventional studies, randomized controlled trials (RCT), non-RCTs, crossover trials, and cohort studies were included, whereas case reports, case-control studies, and reviews were excluded. This review focused on studies that assessed the effects of combining regenerative medicine with exercise therapy, including range of motion, strength, endurance, balance, and aerobic exercises, compared to regenerative medicine alone. Studies that included additional treatments, such as pharmacotherapy, physical modalities, insoles, manual therapy, and patient education, were included if applied to both groups. Primary outcomes were pain and physical dysfunction. Clinical outcomes included pain [visual analog scale (VAS), numerical rating scale (NRS), Likert scale], knee function (strength, range of motion), physical function (such as walking tests, timed up and go test, berg balance test), and patient-reported outcomes [Knee Injury and Osteoarthritis Outcome Score, Western Ontario and McMaster Universities Osteoarthritis Index (WOMAC), New Knee Society Score, Japanese Orthopedic Association (JOA)], measured at least 1-month post-treatment. There were no restrictions on the location, sex, race, country, or language. Non-English language studies were excluded due to limited translation resources.

### Search strategy

The search strategy involved a three-step approach to identify published studies. Initially, a limited search was conducted on MEDLINE (PubMed), CENTRAL, and CINAHL, using relevant articles. Keywords and index terms from the titles and abstracts were used to refine the search strategy for each database. An example of the PubMed search string was:

[“knee osteoarthritis”(MeSH Terms) OR “knee osteoarthritis”(Title/Abstract)] AND (“platelet-rich plasma” OR “stem cell therapy” OR “regenerative medicine”) AND (“exercise therapy” OR “physical therapy” OR “rehabilitation”). The reference lists of the included studies were also screened for additional sources. The complete search strategies for each database are provided in Supplementary Appendix 1. The comprehensive search was conducted on July 27, 2024.

### Selection of studies/sources of evidence

**Figure 1:**
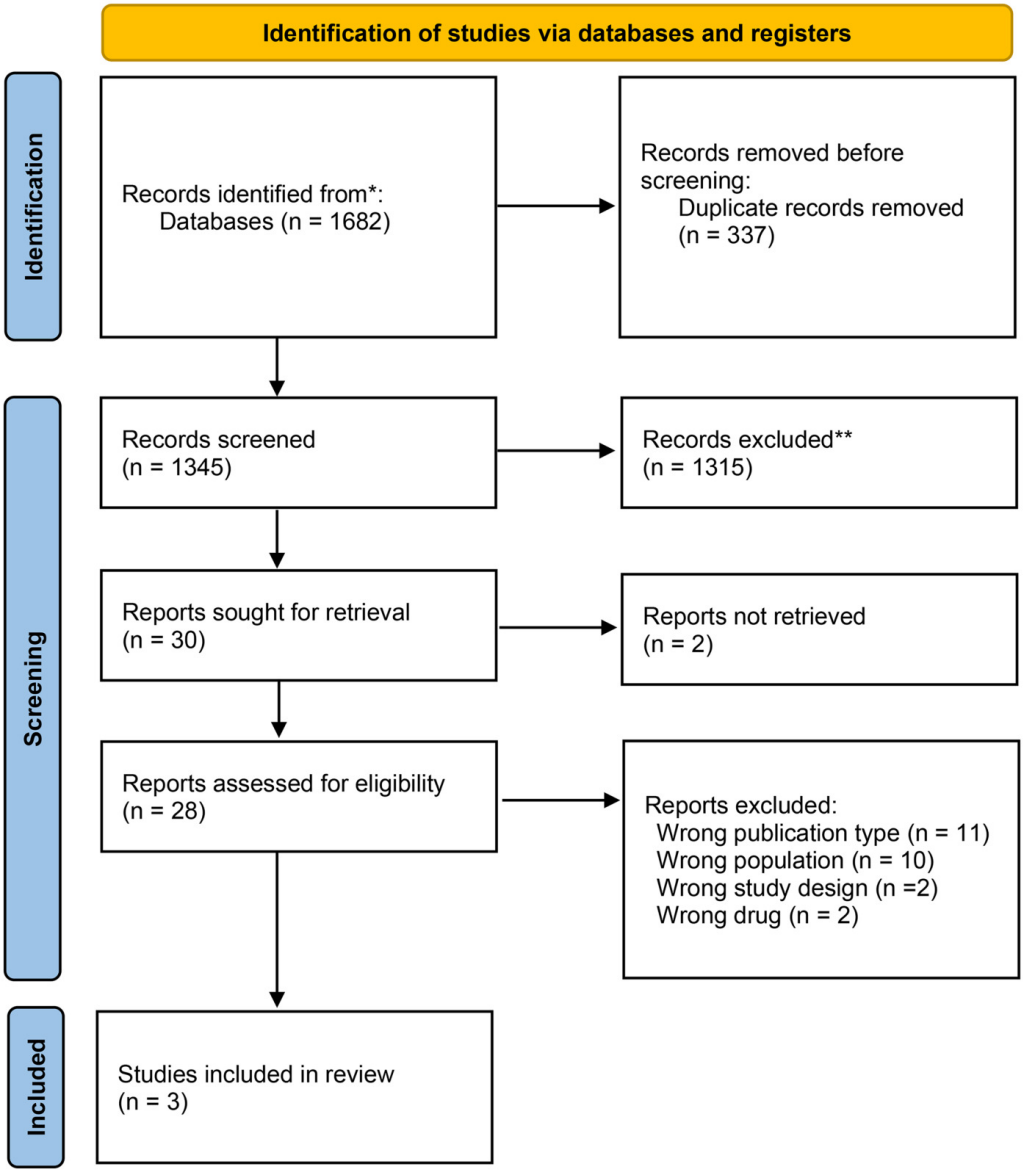
Flowchart of the search strategy and results.

All citations were compiled after the search and duplicates were removed in Rayyan (Qatar Computing Research Institute, Ar Rayyan, Qatar) (19). After a pilot test, two or more independent reviewers assessed the eligibility based on the inclusion criteria by screening titles, abstracts, and full texts. Relevant sources were fully retrieved, and citation details were managed in Rayyan. Reasons for excluding sources were documented and reported. Any disagreement during the selection process were resolved through discussion or an additional reviewer was involved. The PRISMA 2020 flow diagram depicts the search and selection process (17) (Figure 1).

### Data extraction

Data extraction was performed by two or more independent reviewers (T. O. and K. S.) using Microsoft Excel. Information on participants, concepts, context, study methods, and significant findings relevant to the review question were extracted. Detailed extraction forms were provided with information on the authors, authors' countries of origin, study settings, study designs, sample sizes, participants' characteristics (age, sex, and diagnosis), the regenerative medicine used, and clinical outcomes. The draft extraction tool was adjusted and refined as needed. Disagreements were resolved through discussion or by consulting an additional reviewer. Authors were contacted if additional or missing data were required. The extraction sheet included standardized fields such as author, year, country, sample size, study design, regenerative medicine type, exercise intervention details, outcome measures, and follow-up duration to ensure consistency and transparency across studies.

### Risk of bias assessment in the included studies

Two reviewers (T.O. and K.S.) independently evaluated the risk of bias using the Risk of Bias 2 (RoB2) tool for randomized studies and the Risk of Bias in Non-randomized Studies of Interventions (ROBINS-I) tool (20, 21). Disagreements were resolved through discussion or by involving a third reviewer.

## Results

### Search selection

Database searches identified 1,682 titles and abstracts. After removing duplicates, 1,345 articles were screened by titles and abstracts, leaving 30 full-text articles assessed for eligibility. During the second screening, studies were excluded based on specific criteria (Supplementary Appendix 2). Finally, three articles met the inclusion criteria: two studies were RCTs (22, 23), and one was a non-RCT (24).

The detailed selection process is presented in Figure 1 (PRISMA flow diagram).

### Characteristics of included studies

Table 1 summarizes the key characteristics of included studies, including authors, years, the lead author's country, study design, sample size, type of regenerative medicine, participant demographics, and KL grade. Two studies were conducted in Turkey and one in China. The sample sizes ranged from 17 to 32 participants per group.

**Table 1:** Characteristics of included studies.

Author (year)	Country of origin	Study design	Sample size (Int/Ctl)	Types of regenerative medicine	Age (years) (Int/Ctl)	Female (%) (Int/Ctl)	BMI (kg/m^2^) (Int/Ctl)	KL Grade (%) (Int/Ctl)
Argut et al. (21)	Turky	RCT	28/28	PRP	54 ± 7/55 ± 7	71/71	28 ± 4/29 ± 4	II: 36/46 III: 64/54
Bozgeyik et al. (22)	Turky	RCT	17/13	PRP	59.1 ± 6.7/56.2 ± 7.9	100/100	28.7 ± 5.2/28.9 ± 7.6	NR
Yan et al. (23)	China	Non-RCT	32/32	PRP	58.0 ± 6.1/58.1 ± 7.0	47/50	NR	I: 31/31 II: 41/44 III: 28/25

RCT, randomized controlled study; Int, intervention group; Ctl, control group; PRP, platelet-rich plasma; BMI, body mass index; KL grade, Kellgren-Lawrence grade; NR, not reported.

Continuous variables were presented as average ± standard deviations.

### Exercise interventions and outcomes

**Figure 2:**
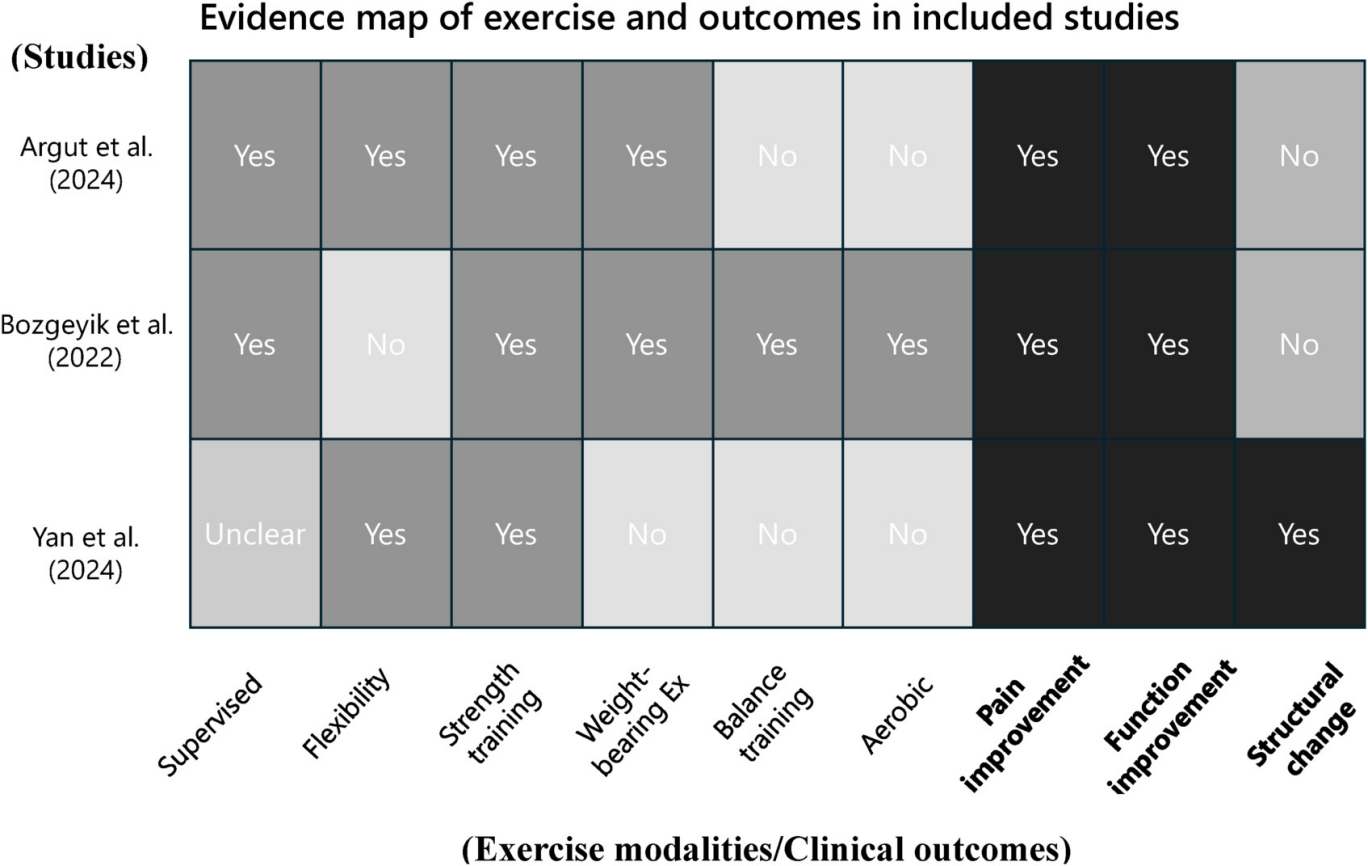
Evidence maps of exercise and outcomes in included studies.

Figure 2 presents an evidence map summarizing the types of exercise interventions and outcome domains assessed in the included studies combining regenerative medicine with exercise therapy. The vertical axis lists the three included studies (22–24), while the horizontal axis categorizes the components into ten elements: supervised exercise, flexibility training, strength training, weight-bearing exercises, balance training, aerobic exercise, pain improvement, functional improvement, structural change, and quality of life.

Supervised exercise sessions were clearly described in two studies (Argut et al. and Bozgeyik et al.), while this information was not specified in Yan et al. (24). Strength training was implemented in all three studies. Flexibility training was used in Argut et al. (22) and Yan et al. (24), whereas balance training and aerobic exercise were reported only in Bozgeyik et al. (23). Weight-bearing exercises were included in Argut et al. (22) and Bozgeyik et al. (23), but not in Yan et al. (24).

Regarding outcome domains, all three studies assessed both pain and functional improvement. However, quality of life was not evaluated in any of the included studies. Structural changes, such as joint effusion, synovial thickness, and cartilage thickness, were assessed only in Yan et al. (24) using musculoskeletal ultrasound.

This evidence map provides a comprehensive visual overview of the current evidence on combined regenerative medicine and exercise therapy for knee osteoarthritis. It reveals heterogeneity in the types of exercise interventions employed and highlights important gaps in the literature. In particular, aerobic and balance exercises have been underutilized, and outcome domains such as structural changes and quality of life remain insufficiently evaluated.

### Conceptual framework of integrated management

**Figure 3:**
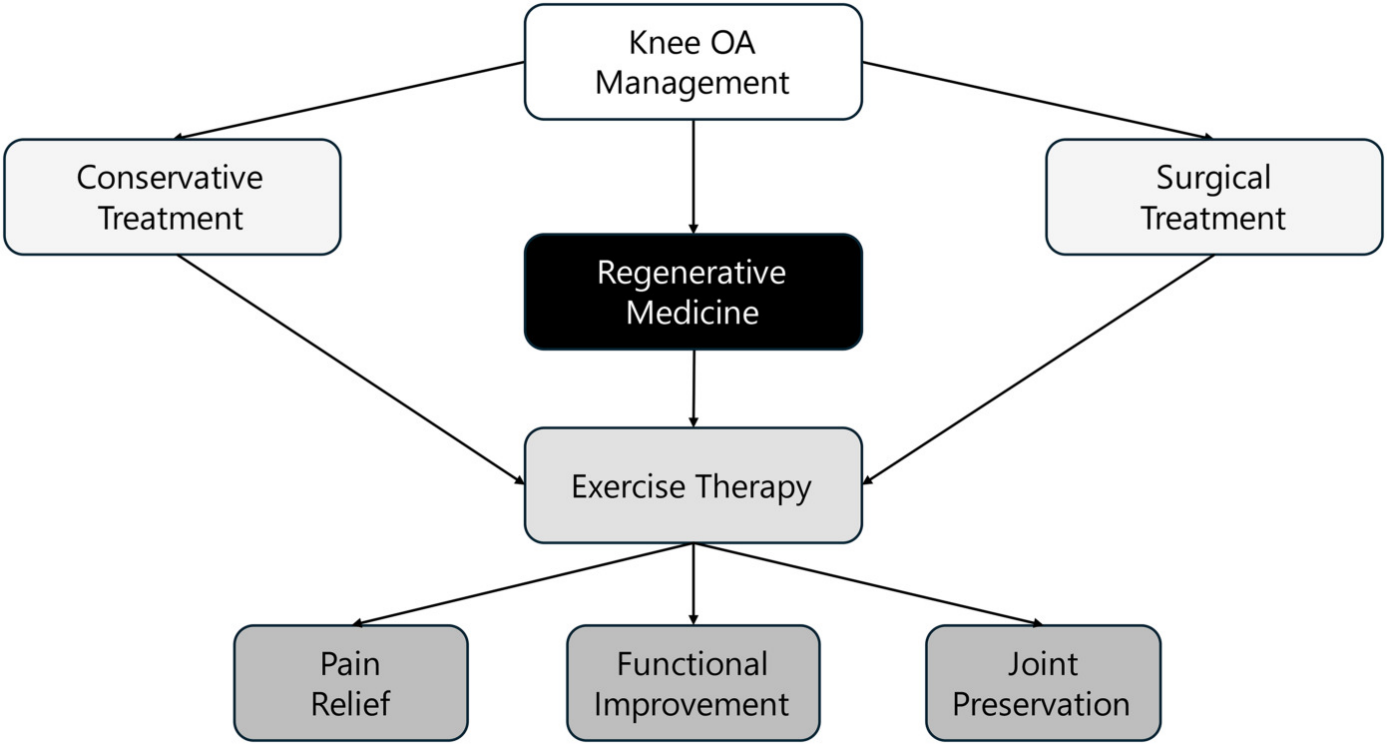
Integrated framework for knee OA management.

To complement the evidence map, Figure 3 provides a conceptual framework that illustrates the integration of regenerative medicine and exercise therapy within the broader landscape of knee OA management. This framework situates the combined approach alongside conservative and surgical treatments and highlights its contribution to key clinical outcomes, including pain relief, functional improvement, and joint preservation.

The figure emphasizes that regenerative therapies serve as a biologically driven intervention, while exercise therapy supports biomechanical and neuromuscular restoration. Together, they function as a bridge between symptom management and structural preservation. This integrated view helps contextualize the role of combination strategies and informs future clinical applications and research directions.

### Risk of bias in studies

**Figure 4:**
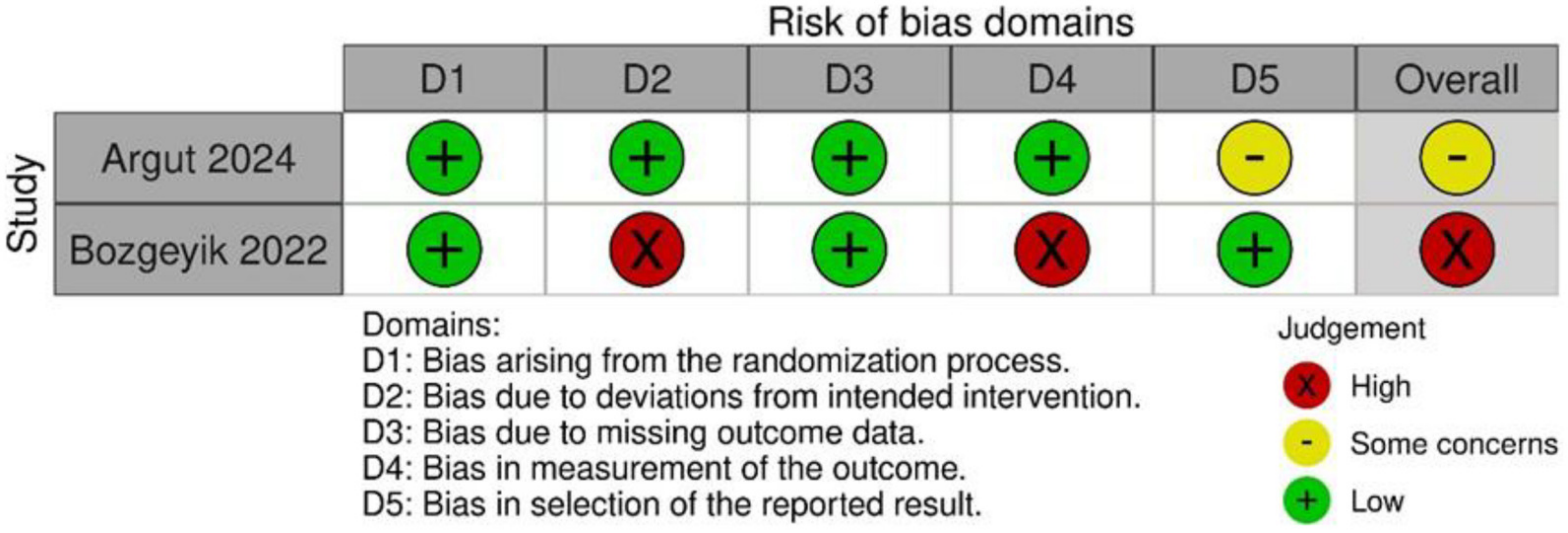
Summary of Cochrane ROB assessments for individual RCT studies.

**Figure 5:**
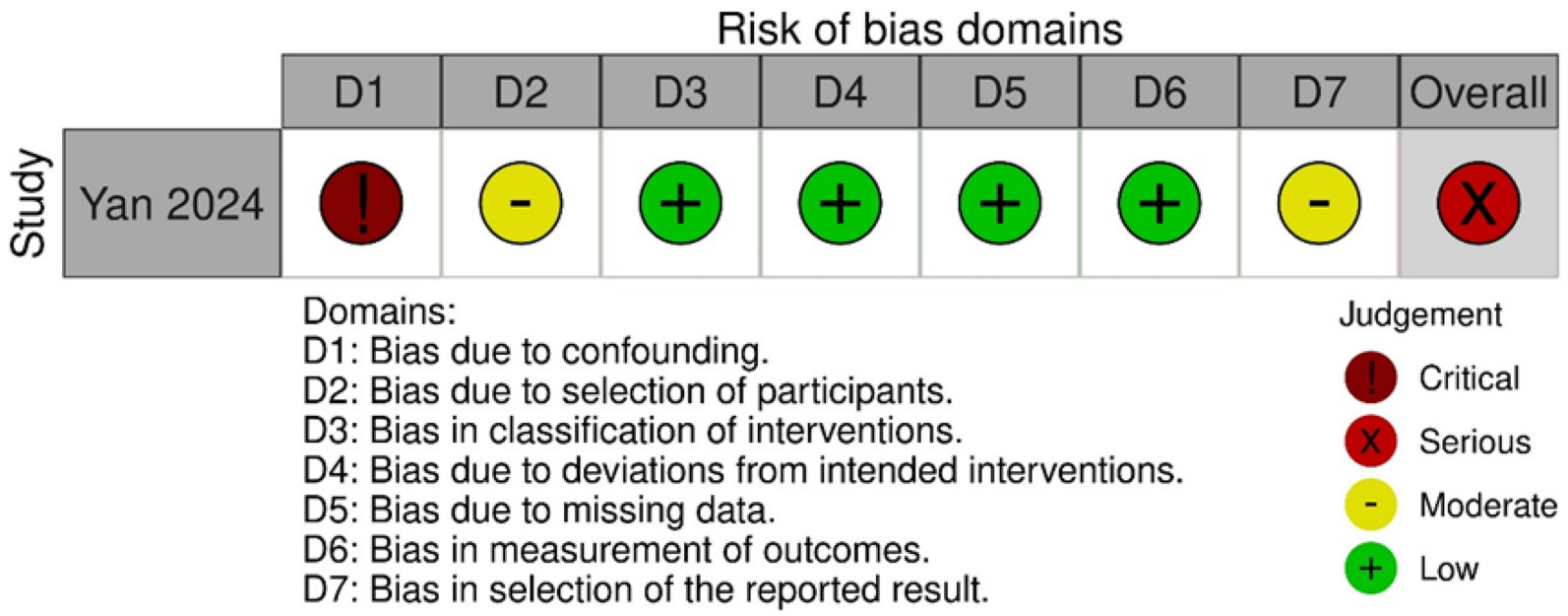
Summary of Cochrane ROBINS-I assessments for individual non-RCT studies.

The results of the RoB2 assessment are shown in Figure 4. Argut et al. (22)'s study demonstrated a low risk of bias in most domains, with minor concerns about the result section, resulting in an overall rating of “some concerns.” In Bozgeyik et al. (23)'s study, most domains indicated a low risk of bias, but deviations from intended interventions and outcome measurement resulted in a high overall risk of bias. Consequently, this study was rated as having a higher overall risk of bias. The ROBINS-I results are shown in Figure 5. The assessment revealed a significant overall risk of bias. Specifically, there was a crucial risk of bias owing to confounding factors and a moderate risk of bias in participant selection. Despite low bias in classifying interventions, deviations from intended interventions, and missing data, moderate concerns remained regarding the selection of reported results. Therefore, although certain aspects had low risk, confounding factors and participant selection concerns contributed to an elevated overall risk of bias.

In summary, Bozgeyik et al. (23), one of the two RCTs, had a high risk of bias due to not using blinding. Yan et al. (24), a non-RCT, was subject to confounding bias, and caution is warranted when interpreting its long-term efficacy.

## Discussion

This scoping review explored the effectiveness of combining exercise therapy with regenerative medicine in patients with knee OA. Across the three included studies, all reported significantly greater improvements in pain and function in the combined intervention groups compared to regenerative medicine alone. These improvements were observed both in the short term (6–24 weeks) and long term (up to 96 weeks), suggesting potential additive or synergistic effects of exercise therapy when used alongside regenerative interventions.

While all studies demonstrated pain reduction, the sustainability and magnitude of the effect varied. For example, Argut et al. (22) reported statistically significant improvements at 24 weeks compared to the control group, though the change may have fallen below the minimal clinically important difference. In contrast, Yan et al. (24) observed consistent pain relief over 96 weeks, supporting the long-term potential of this combined approach. These differences may be attributable to variations in intervention delivery (e.g., supervised vs. home-based), adherence, and study design.

The type and setting of exercise therapy also appeared to affect outcomes. Supervised exercise programs, as used in Argut et al. and Bozgeyik et al., may offer better pain relief and functional improvement than unsupervised or home-based regimens, likely due to improved adherence, feedback, and progression. Supervised training may also help correct movement patterns in real time, further enhancing its effectiveness (25). Future research should aim to standardize exercise dosage, including frequency, intensity, and type, to improve comparability and optimize outcomes. However, Bozgeyik et al. did not find significant improvements in muscle strength, indicating that intensity and specificity of training protocols remain important factors.

Improvements in physical function were reported across all studies, but the interventions differed substantially. Exercise therapy content ranged from flexibility and non-resistive strength exercises to progressive resistance and balance training, with or without supervision. Such diversity limits comparability across studies and may influence the observed effectiveness. Additionally, follow-up durations varied from short-term (6 weeks) to long-term (96 weeks), and study designs included both RCTs and a non-randomized trial, introducing variability in internal validity and risk of bias. These factors should be considered when interpreting the aggregated findings.

Beyond symptom relief, structural improvements were noted. Yan et al. (24) reported positive changes in ultrasound markers, including increased cartilage thickness and reduced joint effusion. Although promising, the clinical significance of these findings remains uncertain. In particular, the increase in cartilage thickness should be further validated in relation to long-term functional improvements and patient-reported outcomes. Additionally, Argut et al. (22) reported improvement in physical but not mental components of health-related quality of life, highlighting a potential need for psychological or behavioral support in OA management. Additionally, Argut et al. (22) reported improvement in physical but not mental components of health-related quality of life, highlighting a potential need for psychological or behavioral support in OA management. Future joint interventions should consider integrating cognitive-behavioral therapy to improve mental health outcomes in patients with knee OA (26).

This review highlights the potential role of platelet-rich plasma (PRP) combined with supervised exercise as a non-surgical, first-line treatment option for patients with moderate knee osteoarthritis (KL grade II–III), particularly those who are unwilling or unsuitable for surgery. These findings offer practical guidance for clinicians seeking effective alternatives to invasive procedures in early- to mid-stage OA management. However, notable inconsistencies across studies should be acknowledged. The included studies varied in regenerative medicine protocols (e.g., pure vs. leukocyte-rich PRP), types and supervision level of exercise interventions (e.g., home-based vs. supervised training), and follow-up durations (ranging from 6 to 96 weeks). These differences may have contributed to variability in treatment outcomes. Moreover, the methodological designs were not uniform; two studies used randomized controlled designs, whereas one employed a non-randomized design, increasing the potential for confounding bias. This heterogeneity limits direct comparisons and underscores the need for more standardized and rigorous research approaches.

This study has several limitations. First, the RoB2 assessments revealed methodological concerns, including selective reporting in Argut et al. (21)'s study and a higher risk of bias in Bozgeyik et al. (22)'s study owing to deviations from intended interventions. These biases should be considered when interpreting the long-term effectiveness of combining regenerative medicine with exercise therapy. Future research should address these issues and explore the mechanisms underlying muscle strength recovery and mental health improvements. Second, the lack of standardization in exercise therapy interventions across studies resulted in variations in exercise type, intensity, and frequency, contributing to inconsistent outcomes in muscle strength and physical function. Standardized protocols are required to draw clear conclusions regarding the most effective exercise components to combine with regenerative medicine. Third, the diversity in study designs is a limitation; whereas two studies used an RCT design, one was nonrandomized, introducing potential confounding and selection biases. Future research should aim for more consistent study designs to strengthen evidence and improve the reliability of findings. Fourth, the number of included studies was small, and all focused exclusively on PRP interventions. Therefore, the effectiveness of combining exercise therapy with other regenerative medicine approaches, such as stem cell therapy, remains unclear and warrants further investigation. Addressing this gap will help broaden the applicability of findings and inform future treatment strategies.

## Conclusions

This scoping review suggests that combining regenerative medicine with exercise therapy provides significant short-term pain relief and maintains functional improvement over the long term in patients in knee OA patients. However, inconsistent effects on muscle strength and mental health indicate the need for more targeted exercise interventions. Exercise programs should follow the FITT (frequency, intensity, time, and type) principle, beginning with non-weight-bearing exercises in the early phase and gradually progressing to resistance training. In addition, incorporating cognitive-behavioral therapy may help address mental health needs. High-quality randomized trials with standardized protocols are essential to confirm the effectiveness of this combined approach and to optimize its clinical application.

## Data Availability

The original contributions presented in the study are included in the article/Supplementary Material, further inquiries can be directed to the corresponding author.
