# Supplementary material for: Effectiveness of combined regenerative medicine and exercise therapy for patients with knee osteoarthritis: a scoping review
Source: Front Rehabil Sci. 2025 Jul 22;6:1612615. doi: 10.3389/fresc.2025.1612615 (PMC12321541; doi:10.3389/fresc.2025.1612615)
Supplement: Supplementary file 1 [file Datasheet1.pdf]

Supplementary File: Prompts Used with Generative AI

- AI Tool Used

  Name: ChatGPT

  Model: GPT-4

  Provider: OpenAI

  Accessed: May 2025

- Purpose of Use

  ChatGPT was used solely to assist in editing the manuscript for clarity and grammar. No scientific content, data analysis, or interpretation was generated by AI.

- Initial Prompt

  Please help me improve the clarity and coherence of the introduction section of this scoping review manuscript. Focus on grammar, fluency, and logical flow without altering the meaning.

- Final Prompt

  Please polish the revised Discussion section of the manuscript to ensure academic tone and consistency, and improve transitions between paragraphs. Do not change any scientific content.
