# Supplementary material for: Effectiveness of combined regenerative medicine and exercise therapy for patients with knee osteoarthritis: a scoping review
Source: Front Rehabil Sci. 2025 Jul 22;6:1612615. doi: 10.3389/fresc.2025.1612615 (PMC12321541; doi:10.3389/fresc.2025.1612615)
Supplement: Supplementary file 2 [file Datasheet2.pdf]

Appendix 2. Reports excluded from this review and the corresponding reasons for exclusion

Reason for exclusion	References
Intervention not involving recognized regenerative medicine	Elgendy MH, Elsamahy SA, Mostafa MSEM, Hamza MSK. Efficacy of shockwave therapy versus intra-articular platelet-rich plasma injection in the management of knee osteoarthritis: a randomized controlled trial. International Journal of Pharmaceutical Research. 2020; 12(4): 4283-4289.
Intervention not involving recognized regenerative medicine	Sert AT, Sen EI, Esmaeilzadeh S, Ozcan E. The effects of dextrose prolotherapy in symptomatic knee osteoarthritis: a randomized controlled study. J Altern Complement Med. 2020; 26(5): 409-417.
No combination of regenerative medicine and exercise therapy assessed	Rezasoltani Z, Azizi S, Najafi S, Sanati E, Dadarkhah A. Physical therapy, intra-articular dextrose prolotherapy, botulinum neurotoxin, and hyaluronic acid for knee osteoarthritis: randomized clinical trial. Int J Rehabil Res. 2020; 43(3): 219-227.
No combination of regenerative medicine and exercise therapy assessed	Mardani-Kivi M, Leili EK, Esnaashari S, Hashemi-Motlagh K, Azari Z. Relationship between the underlying factors and the treatment results of platelet-rich plasma (PRP) injection in degenerative knee disease: a blinded randomized study. Asian J Sports Med. 2023;14(1):1.
No combination of regenerative medicine and exercise therapy assessed	Buendía-López D, Medina-Quirós M, Fernández-Villacañas Marín MA. Clinical and radiographic comparison of a single LP-PRP injection, a single hyaluronic acid injection and daily NSAID administration with a 52-week follow-up: a randomized controlled trial. J Orthop Traumatol. 2018; 19(1): 3.
No combination of regenerative medicine and exercise therapy assessed	Rayegani SM, Raeissadat SA, Taheri MS, Babaee M, Bahrami MH, Eliaspour D, et al. Does intra articular platelet rich plasma injection improve function, pain and quality of life in patients with osteoarthritis of the knee? A randomized clinical trial. Orthop Rev (Pavia). 2014; 6(3): 5405.
No combination of regenerative medicine and exercise therapy assessed	Güvendi EU, Aşkin A, Güvendi G, Koçyiğit H. Comparison of Efficiency Between Corticosteroid and Platelet Rich Plasma Injection Therapies in Patients With Knee Osteoarthritis. Arch Rheumatol. 2017; 33(3): 273-281.
No combination of regenerative medicine and exercise therapy assessed	Angoorani H, Mazaherinezhad A, Marjomaki O, Younespour S. Treatment of knee osteoarthritis with platelet-rich plasma in comparison with transcutaneous electrical nerve stimulation plus exercise: a randomized clinical trial. Med J Islam Repub Iran. 2015: 29: 223.

No combination of regenerative medicine and exercise therapy assessed	Hedayati, R, Aminian-Far, A, Darbani, M, Dadbakhsh M, Ehsani F. Efficacy of glucosamine compounds phonophoresis in knee osteoarthritis. Koomesh. 2016; 18(2): 276-285.
No combination of regenerative medicine and exercise therapy assessed	Dumais R, Benoit C, Dumais A, Babin L, Bordage R, de Arcos C, et al. Effect of regenerative injection therapy on function and pain in patients with knee osteoarthritis: a randomized crossover study. Pain Med. 2012; 13(8): 990-999.
No combination of regenerative medicine and exercise therapy assessed	Akan O, Sarikaya NO, Kocyigit H. Efficacy of platelet-rich plasma administration in patients with severe knee osteoarthritis: can platelet-rich plasma administration delay arthroplasty in this patient population? Int J Clin Exp Med. 2018; 11(9): 9773-9483.
No combination of regenerative medicine and exercise therapy assessed	Alcin, FN, Turhan, B. Comparison of the effects of a physiotherapy protocol and platelet-rich plasma treatment on pain, functionality, and kinesiophobia in women with medial compartment osteoarthritis of the knee. Physiotherapy Quarterly. 2023; 31: 39-45.
Narrative review	O'Connell B, Wragg NM, Wilson SL. The use of PRP injections in the management of knee osteoarthritis. Cell Tissue Res. 2019; 376(2): 143-152.
Narrative review	Kon E, Filardo G, Drobnic M, Madry H, Jelic M, van Dijk N, et al. Non-surgical management of early knee osteoarthritis. Knee Surg Sports Traumatol Arthrosc. 2012; 20(3): 436-449.
Not a peer-reviewed article	Wang H, Sun W, Zhao D. Platelet-rich plasma combined with exercise therapy: A treatment option for knee osteoarthritis. Asian J Surg. 2023; 46(3): 1382-1383.
Not a peer-reviewed article	Baysal E, Budak M, Atilgan E, Tarakci D. Comparison of efficacy of different rehabilitation approaches in individuals with knee osteoarthritis. Annual European Congress of Rheumatology. 2019; 78(2): 2155.
Not a peer-reviewed article	Argut SK, Celik D, Ergin O, Kilicoglu O. THE COMPARISON OF PRP ALONE, SUPERVISED EXERCISE ALONE, AND PRP COMBINED WITH SUPERVISED EXERCISE IN MANAGEMENT OF KNEE OSTEOARTHRITIS. Annals of the Rheumatic Diseases. 2023; 82. 1818.
Not a peer-reviewed article	Raeissadat SA, Ghorbani E, Sanei M, Rayegani SM, Babaee M, Soleimani R. Does platelet rich plasma change the volumetric findings of mri imaging? (a randomised clinical trial). Annals of the Rheumatic Diseases. 2018; 77: 1610-1611.
Not a peer-reviewed article	Works C, Miller J. Is prolotherapy effective in reducing pain and improving function in patients with knee OA? Evid Based Pract. 2020; 23(3): 19-20.

Not a peer-reviewed article	Raeissadat SA, Ghorbani E, Rayegani SM, Sanei Taheri M, Babaee M, Soleimani R. Volumetric findings of MRI after platelet rich plasma injection in knee osteoarthritis (A randomized clinical trial). Annals of Physical and Rehabilitation Medicine 61S. 2018; e9: ISPR8-1547.
Not a peer-reviewed article	Hebei Medical University Third Hospital. The Efficacy of PRP Combined With Exercise to Treat Knee Osteoarthritisq. 2022. ClinicalTrials. gov NCT05585216.
Not a peer-reviewed article	Argut SK. Exercise and PRP vs Exercise Alone in Patients With Knee Osteoarthritis. ClinicalTrials. gov NCT04697667.
Not a peer-reviewed article	Riphah International University. Mulligan and Maitland Techniques in Post PRP Knee Osteoarthritis. ClinicalTrials. gov NCT06460168.
Not a peer-reviewed article	Khasru MR. Adipose-tissue-derived Total-Stromal-cells (TOST) Therapy in Knee Osteoarthritis. ClinicalTrials. gov ID: NCT05280002.
Not a peer-reviewed article	ÖZTÜRK MU. Different Concentrations of Dextrose Prolotherapy Treatment in Knee Osteoartritis ClinicalTrials. gov NCT05537077.
Unable to obtain full text	BozgeyIk S, Kilinc H, HurI G, Guney Deniz H. The comparison of three different management strategies following platelet rich plasma injection in patients with knee OA. Annals of the Rheumatic Diseases. 2023; 82: 1830-1831.
Unable to obtain full text	Akan, O, Olmez Sarikaya, N, Kocyigit, H. Efficacy of platelet rich plasma (prp) in severe knee osteoarthritis: can PRP injections delay arthroplasty? International Journal of Rheumatic Diseases. 2017; 28: S552.
